# Flow-Induced Friction Reduction in Shear Flows of Unentangled Polymer Melts

**DOI:** 10.1021/acs.macromol.6c01365

**Published:** 2026-07-23

**Authors:** Christos Psevdos, Giovanni Ianniruberto

**Affiliations:** Department of Chemical, Materials, and Production Engineering, 9307 Federico II University, Piazzale Tecchio 80, Napoli 80125, Italy

## Abstract

We here analyze very recent shear flow data of three unentangled polystyrene (PS) melts with different molar masses *M* (*Macromolecules* **2025**, 58, 7062–7083) through single-chain Brownian dynamics simulations that account for finite chain extensibility and flow-induced friction reduction. It is confirmed here that in PS melts the monomeric friction coefficient ζ must significantly decrease as the Kuhn segment order parameter *S* increases, as also shown by existing many-chain molecular dynamics simulations. The resulting function ζ­(*S*) is almost indistinguishable for the two higher *M* samples and comparable to that previously extracted from uniaxial extension data of a PS melt with a similar *M* (*Macromolecules* **2019**, 52, 4610–4616). A weaker dependence of ζ on *S* is, however, observed for the lowest *M* melt. This discrepancy, combined with the fact that (contrary to data) our single-chain simulations predict *N*_2_ ≈ 0, suggests the presence of many-chain effects other than just the ζ­(*S*) dependence. The fact that single-chain models still miss some physics is confirmed by the successful comparison between PS melt data and existing many-chain simulations. The agreement is, however, limited to shear flows since the same comparison fails in elongational flows.

## Graphical abstract

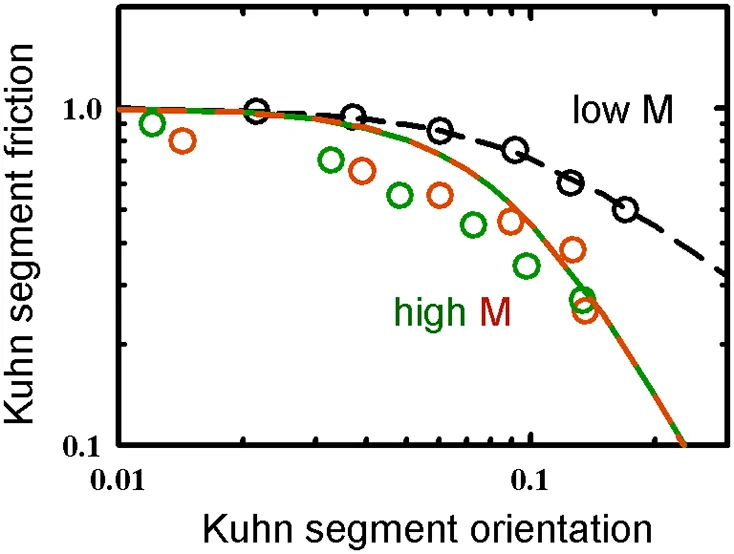

## 1 Introduction

In recent times, there has been a renewed interest in low-molar-mass polymer melts thanks to the intriguing experimental results in fast shear and extensional flows provided by several authors.^1–4^ Unentangled polystyrene (PS) melts exhibit an unexpectedly complex behavior in fast shear flows.^1,2,4^ The steady-state viscosity significantly decreases with increasing shear rate (shear thinning), and a nonmonotonic behavior (overshoots and undershoots) is observed during the transient startup. All these phenomena are well documented for entangled melts,^5,6^ and only recently have they been systematically investigated in unentangled ones.^1–4^ Shear flows of unentangled PS melts also give rise to first as well as second normal stress differences, *N*_1_ and *N*_2_,^4^ with a ratio *N*_1_/*N*_2_ comparable in magnitude to that observed in entangled polymers.^7,8^

Complex nonlinear phenomena are also observed in uniaxial extensional flows of unentangled melts, with a steady-state viscosity that changes in a nonmonotonic way when increasing the stretch rate: an initial increase after the Newtonian plateau is followed by a drastic decrease of the viscosity.^3^ This nonmonotonic response is usually not observed in entangled polymers that directly transition to the thinning regime after the Newtonian plateau.^9,10^

The molecular origin of the nonlinear phenomena described above is still under investigation. The classical bead–spring molecular model, known as the Rouse model, though consistent with rheological data in the linear range,^11^ completely fails in capturing the nonlinear response.^12^ As is well known, in shear flows the Rouse model predicts a constant steady viscosity, a zero second normal stress difference, and a monotonic transient response during startup. In steady uniaxial extensional flows, the Rouse model predicts a diverging viscosity at the onset of the nonlinear range, with no evidence of thinning.

The failure of the Rouse model in both shear and extensional flows is partly due to the infinite extensibility of the harmonic springs. Attempts to quantitatively describe experiments on unentangled PS melts have clearly shown that finite extensibility (FENE) effects alone are not enough.^13,14^ In shear, FENE effects promote chain shrinking in the velocity gradient direction, thus inducing thinning, which is, however, much weaker than observed.^14^ In steady uniaxial extension, the divergence predicted by the Rouse model is suppressed and replaced by a second plateau, without, however, any thinning.^13^

Colby and co-workers^15^ attributed the shear thinning of unentangled melts to the “freezing” of the Rouse modes slower than the flow (i.e., those with relaxation times longer than the reciprocal shear rate, γ*˙*), which are assumed not to contribute to viscous dissipation. Dalne et al.^4^ adapted this so-called shear blob model to transient shear flows as well, providing good agreement with shear startup viscosity data. This model (also named the shear slit model by Parisi et al.^16^ as well as by Dalne et al.^4^ themselves) cannot easily be extended, however, to extensional flows.

As suggested in several papers by Ianniruberto, Marrucci, and coworkers,^13,14,17^ thinning in polymer melts may be due to the flow-induced reduction of the monomeric friction coefficient: under fast flows, monomers co-align, and their mobility is enhanced. This is clearly confirmed by several nonequilibrium molecular dynamics simulations measuring chain mobilities under flow and/or deformation.^17–20^ A flow-induced increase of the mobility implies a decrease of the viscosity. This phenomenon is relevant in both shear and extensional flows, provided that they are fast enough to induce chain stretch.

Motivated by the recent paper by Dalne et al.,^4^ reporting a systematic experimental campaign on three unentangled PS melts of different molar masses in shear flows, we here attempt to quantitatively describe their complex rheological behavior in steady shear by resorting to single-chain Brownian dynamics simulations. In particular, we aim to explore the role of the molecular mechanisms mentioned above (finite-chain extensibility, flow-induced reduction of the monomeric friction coefficient).

This paper is organized as follows. After recalling the Brownian dynamics simulation methodology, we compare predictions to the data of Dalne et al. Then, we attempt a quantitative comparison between the data of Dalne et al. and existing nonequilibrium molecular dynamics simulations^21^ to investigate the importance of many-chain effects. Perspectives of future work conclude this paper.

## 2 Simulation Methodology

We model unentangled polymer melts by resorting to single-chain Brownian dynamics simulations, where polymers are bead–spring chains, coarse-grained at the Kuhn segment level. In particular, we use Fraenkel chains^22^ made up of beads endowed with a friction coefficient ζ and connectors of root-mean-square length *l*_K_ having a suitably large spring constant *H* that ensures the inextensibility of the contour length of the chain.

The model makes use of the following equations:

$$F=H\left(l-l_{K}\right)u$$ 1

$$\zeta \left(\frac{dR_{i}}{dt}-\kappa \cdot R_{i}\right)=F_{i+1}-F_{i}+f_{i},,F_{0}=F_{N_{K}+1}=0$$ 2

$$\sigma =\nu \langle \overset{N_{K}}{\underset{i=1}{\sum }}F_{i}Q_{i}\rangle$$ 3

where ***F*** is the Fraenkel spring force in a chain segment with orientation ***u*** and *l* is the current segment length. Equation 2 is the Langevin equation for the position ***R**_i_* of the *i*-th bead (0 ≤ *i* ≤ *N_K_*), where ***κ*** is the velocity gradient, ***F**_i_* obeys eq 1 (for 1 ≤ *i* ≤ *N_K_*), and ***f**_i_* is the Brownian force with zero mean and variance ⟨ ***f**_i_* (*t*) ***f**_i_* (*t*′)⟩ = 2ζ*k*_B_ *T*δ­(*t* – *t*′) ***I***, consistent with the fluctuation–dissipation theorem. Angle brackets ⟨···⟩ denote the ensemble average, *k*_B_ *T* denotes the thermal energy, δ(·) denotes the Dirac delta function, and ***I*** denotes the unit tensor. Equation 3 is the stress tensor expression, with ν being the number density of chains and ***Q**_i_* ≡ ***R**_i_* – ***R***_*i*–1_ = *l_i_ **u**_i_* being the vector connecting consecutive beads.

Equations 1–3 contain five material-dependent parameters, i.e., *H*, *l_K_*, ζ, *N_K_,* and *ν*. The chain number density ν is obviously given by $\nu =N_{A}\rho /M$ , with $N_{A}$ being the Avogadro number, ρ being the polymer density, and *M* being the polymer molar mass. The number *N_K_* of Kuhn segments is given by *N_K_* = *M*/*M_K_*, where *M_K_* is the Kuhn segment molar mass, equal to 0.9 kDa for PS.^3^ The other three parameters do not depend on *M*. The Kuhn length *l*_K_ is chemistry-dependent and obviously proportional to *M_K_*.^11^ The spring constant *H* must be large enough to guarantee bond inextensibility, as mentioned above. Its specific value only affects the short-time (or high-frequency) response,^20^ certainly not relevant in the steady-state results examined here. The only real parameter of the simulations is the bead friction coefficient ζ. We determine its equilibrium value ζ_eq_ by fitting the zero-shear viscosity, as detailed in the next section. Needless to say, the model equations can be run with the equilibrium value of the monomeric (or bead) friction coefficient ζ_eq_ also far from equilibrium. For such a case, the model only accounts for the inextensibility of the contour length of the polymer chain.

## 3 Data vs Single-Chain Simulations

### 3.1 Linear Viscoelasticity

Figure 1 shows linear viscoelastic data of the three unentangled PS melts with *M* ≈ 10, 20, and 30 kDa (referred to as PS10k, PS20k, and PS30k, respectively) as reported by Dalne et al.^4^ Lines in Figure 1 are predictions obtained by assuming that the linear relaxation modulus G­(*t*) is the sum of two contributions:

4 $$G\left(t\right)=G_{g}\left(t\right)+G_{R}\left(t\right)$$

**Figure 1:**
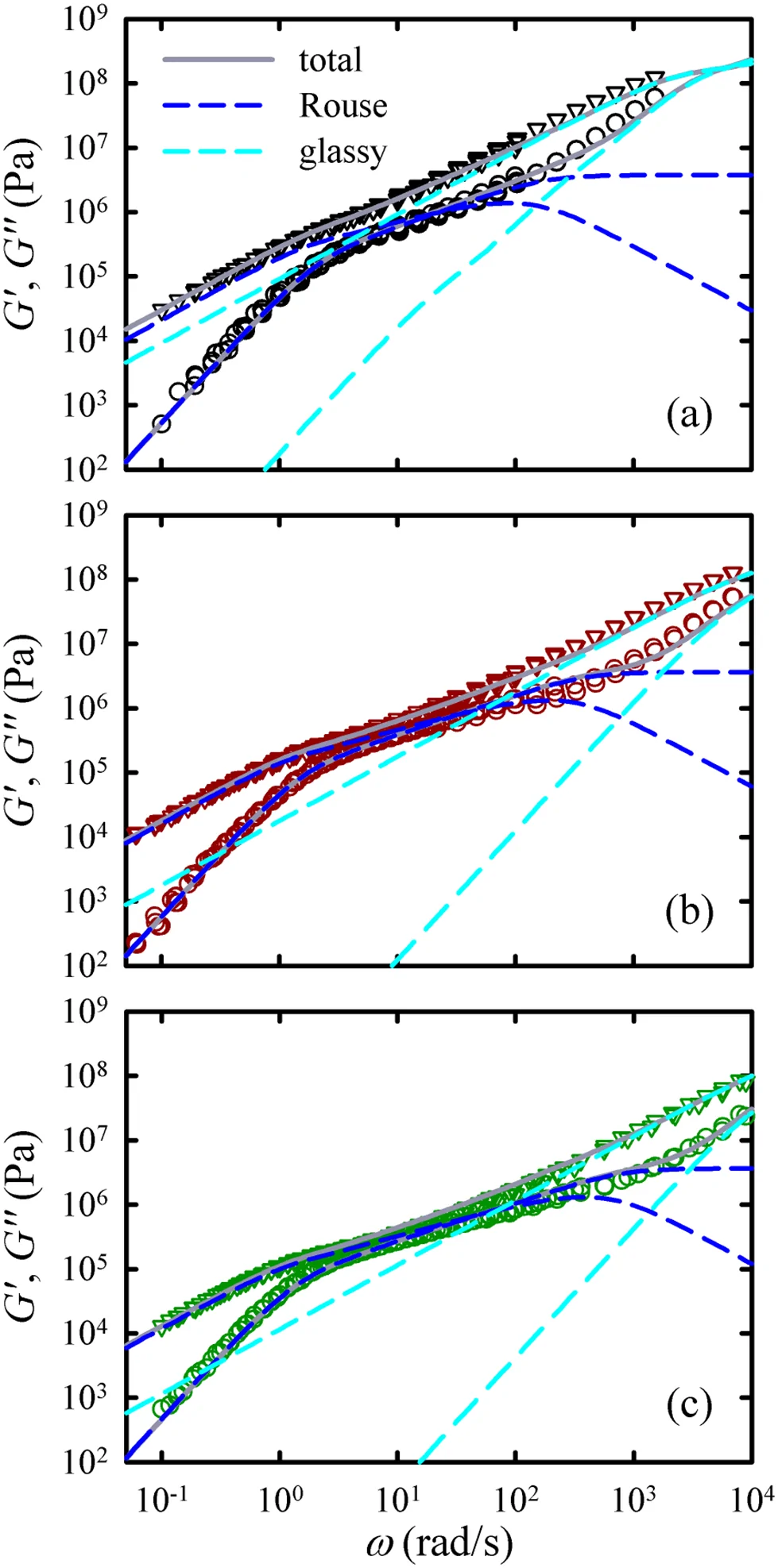
Frequency response of PS10k at 115 °C (a), PS20k at 125 °C (b), and PS30k at 130 °C (c). Symbols represent *G*′, *G*″ data from Dalne et al.^4^ Blue and cyan curves denote the Rouse and glassy contributions, respectively, with gray lines representing their sum.

The first contribution, *G_g_* (*t*), accounts for the glassy relaxation, here taken from a multimode Maxwell fit of the high-frequency response. The high-frequency range is the domain in which the data of the three samples coincide if plotted at the same distance from the glass-transition temperature, *T*_g_. Table 1 reports the glassy modes {τ_*g*,*p*_,*G*_*g*,*p*_} at *T* = 125°C, obtained by fitting the high-frequency response of PS20k. Those modes are then shifted to the desired temperatures by using the Williams–Landel–Ferry equation with the parameters reported by Dalne et al.^4^

**Table 1:** Glassy Modes of PS20k at 125 °C

τ_g_ (s)	*G_g_* (MPa)
2.9 × 10^–6^	5.3 × 10^2^
1.2 × 10^–5^	4.2 × 10^2^
5.3 × 10^–5^	1.9 × 10^2^
2.2 × 10^–4^	3.8 × 10^1^

The second contribution in eq 4 accounts for the rubbery dynamics, assumed to obey the Rouse model, i.e., $G_{R}\left(t\right)=\nu k_{B}T\overset{N_{K}}{\underset{i=1}{\sum }}e^{-t/\tau_{i}}$ , with τ_i_ = τ*_R_*/*i* ^2^ and $\tau_{R}=\frac{\zeta_{eq}l_{K}^{2}}{24k_{B}T}\sin^{-2}\left[\frac{\pi N_{K}}{2\left(N_{K}+1\right)}\right]$ . The friction coefficient at equilibrium, ζ_eq_, is then determined by matching the zero-shear viscosity, $\eta_{0}=\eta_{g}+\eta_{R}=\underset{p}{\sum }\tau_{g,p}G_{g,p}+\nu k_{B}T\underset{i}{\sum }\tau_{i}$ , with the experimental value. The Rouse times τ*_R_* so obtained are reported in Table 2, together with the Rouse times determined by Dalne et al.^4^ Results are consistent, as expected.

**Table 2:** Rouse Times of the PS Melts

Sample	*T* (°C)	τ_*R*,exp_ (s)	τ*_R_* (s)
PS10k	115	0.42	0.41
PS20k	125	0.56	0.57
PS30k	130	0.65	0.62

It is fair to note that if the Rouse times reported in Table 2 are shifted at the same distance from *T_g_*, they do not accurately obey the expected *M* ^2^ scaling predicted by the Rouse theory. This is not due to a failure of the Rouse model but rather to a failure of the time−temperature superposition principle in the glassy-to-rubbery transition zone, as already emphasized by Dalne et al.^4^

### 3.2 Steady Shear

Figure 2 reports the steady-shear viscosity of the three PS melts examined by Dalne et al.,^4^ together with our predictions obtained by summing the Fraenkel chain contribution with ζ = ζ_eq_ to the glassy one determined above. The finite extensibility of the Fraenkel chains introduces shear thinning, which is, however, modest compared to the data, as already noted by Ianniruberto and Marrucci.^14^

**Figure 2:**
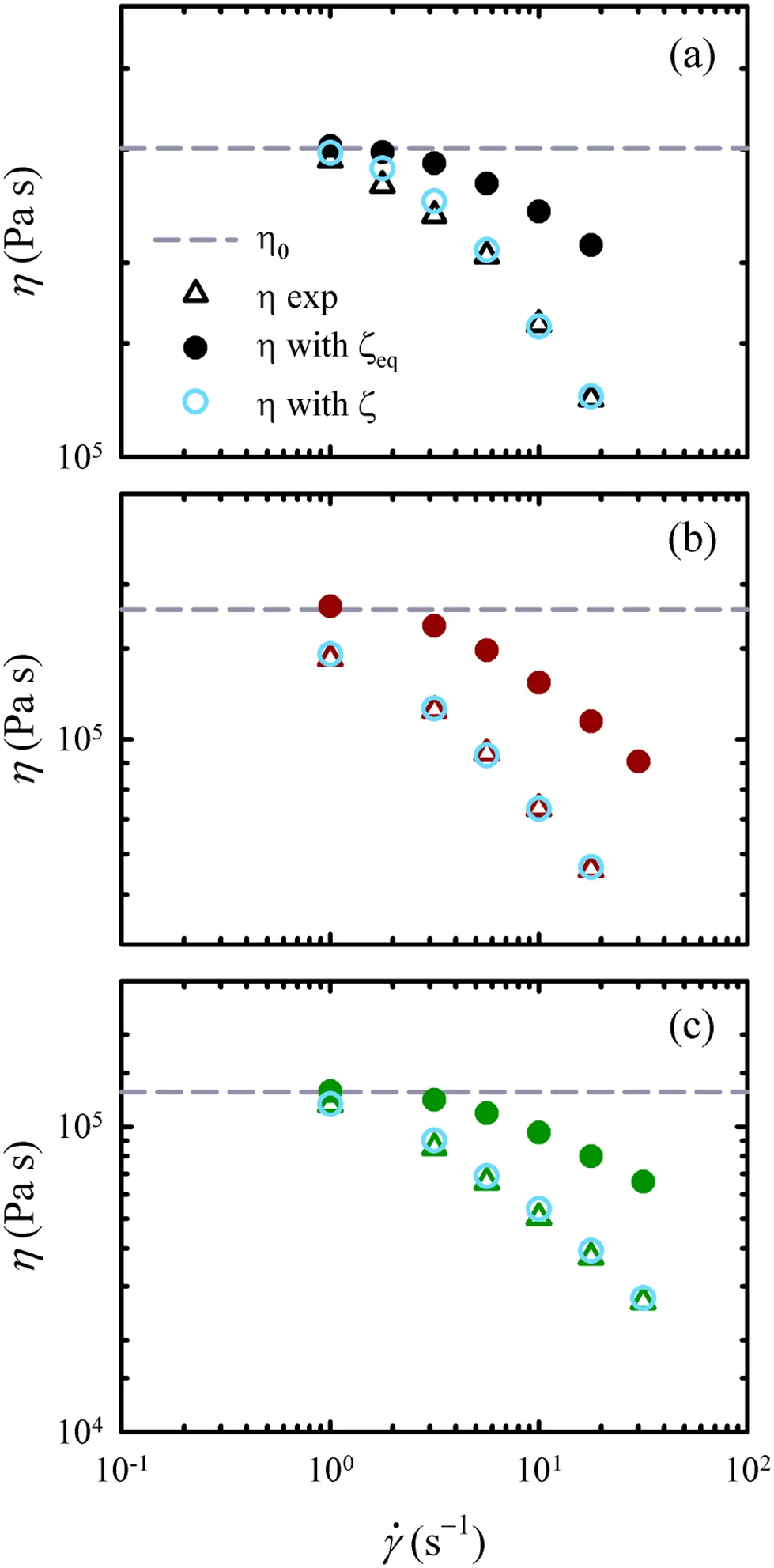
Steady-shear viscosity of PS10k at 115 °C (a), PS20k at 125 °C (b), and PS30k at 130 °C (c). Triangles indicate data from Dalne et al.^4^ Filled circles are predictions with the equilibrium friction coefficient ζ_eq_; empty circles are obtained by adjusting the friction coefficient ζ. Dashed lines represent the experimental zero-shear viscosities.

Normal stress differences were measured by Dalne et al.^4^ only for PS20k and PS30k. In our simulations, the shear thinning of the first normal stress coefficient $\psi_{1}=N_{1}/{\gamma ̇}^{2}$ is again modest compared to the data (results not shown), while the second normal stress difference *N*_2_ is predicted to be zero, contrary to data showing −*N*_2_/*N*_1_ ≈ 0.3 (at the explored shear rates). The vanishing of *N*_2_ comes as no surprise in Rouse-like models (as opposed to tube models):^12^ it is typical of single-chain models that do not introduce any chain anisotropy between the velocity gradient and vorticity directions.

As mentioned in the Introduction, the extra shear thinning shown by data can be attributed to a flow-induced reduction of the monomeric friction coefficient, ζ. Similarly to Ianniruberto and Marrucci,^14^ we determine here, by a trial-and-error procedure, the value of ζ needed to match the steady-shear viscosity at each shear rate γ̇ and for all three samples. Inspired by the existing literature on the stress-induced acceleration of the glassy dynamics,^23^ we have arbitrarily assumed here that the glassy times in Table 1 reduce proportionally to ζ/ζ_eq_, as done previously.^13,14,24^ This premature thinning of the glassy contribution is consistent with the observations of Matsumiya et al.^25^ on styrene oligomer melts in steady shear flows.

Figure 2 shows the quality of the fit for all samples. In Figure 3, we report the fitted ζ values as a function of the Rouse-time-based Weissenberg number *Wi_R_* = γ̇τ*_R_* (panel a) and of the Kuhn segment order parameter *S* (panel b). The latter is given by^26^

$$S=\sqrt{{\left(\left\langle u_{x}^{2}\right\rangle -\left\langle u_{y}^{2}\right\rangle \right)}^{2}+4\langle u_{x}{u_{y}\rangle }^{2}}$$ 5

where *u_x_* and *u_y_* are the components in the shear and gradient directions, respectively, of the Kuhn segment orientation vectors. Obviously, the order parameter *S* is zero at equilibrium, while approaching unity when chains become increasingly oriented in the shear direction. The ensemble average in eq 5 is made over all Kuhn segments, irrespective of their position along the chain, hence ignoring differences in orientation between “inner” and “outer” Kuhn segments, and the possible role of nematic interactions between them.

**Figure 3:**
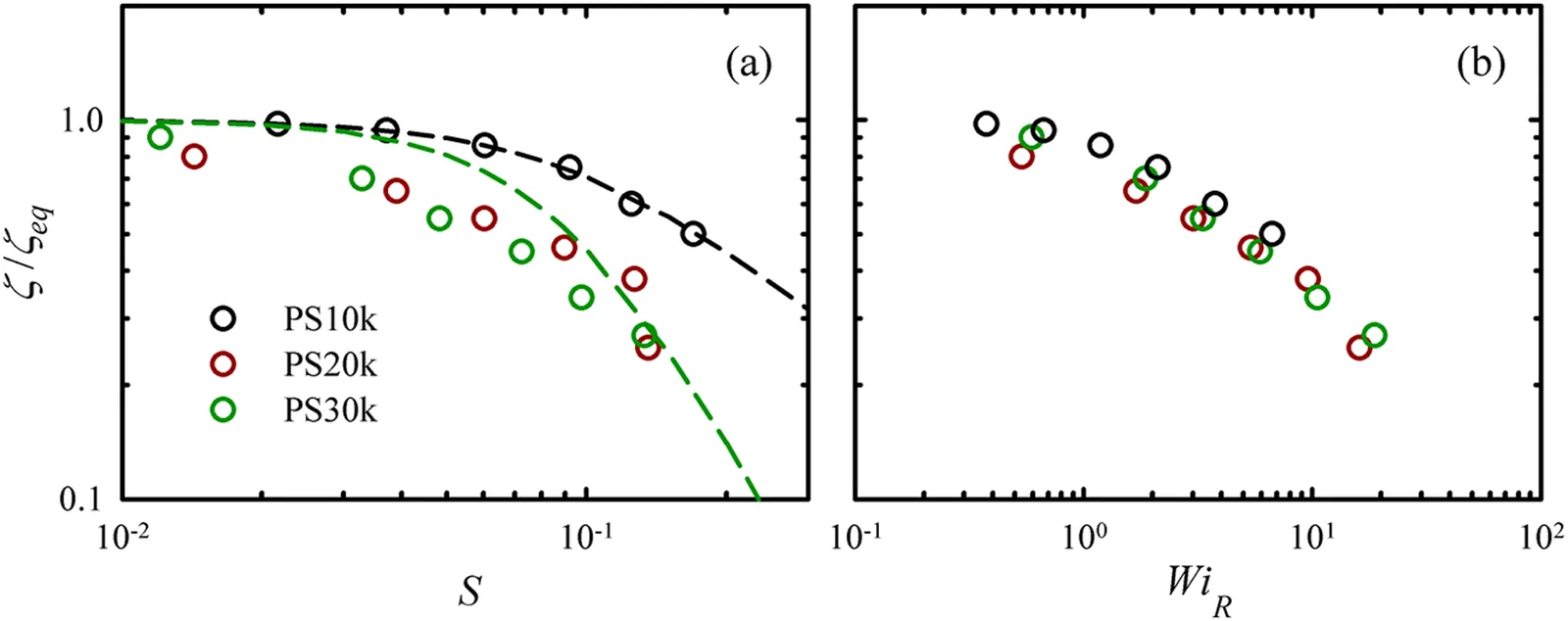
Normalized Kuhn segment friction coefficients required to fit the steady-shear viscosity data of Figure 2, plotted against the Kuhn segment order parameter (a) and the Weissenberg number (b). Black and green dashed lines in panel (a) represent the empirical ζ­(*S*) functions fitted for PS13k in shear^14^ and PS27k in uniaxial elongation,^13^ respectively.

As expected, starting from the equilibrium value ζ_eq_ in slow flows, ζ decreases with increasing shear rate because of an increasing monomer (i.e., Kuhn segment) coalignment. Though the results for the ratio ζ/ζ_eq_ nicely collapse for all three samples when plotted as a function of *Wi_R_* (panel b), they are significantly “separated” for PS10k when plotted vs the Kuhn segment order parameter *S* (panel a). This comes as a surprise since bead friction is a local (i.e., Kuhn segment scale) property, expected to be independent of the polymer molar mass.

In Figure 3a, we also report the friction reduction laws used in previous papers.^13,14^ The black dashed line represents the friction-reduction law extracted from the steady-shear data of Santangelo and Roland,^2^ referring to an unentangled PS melt with *M* ≈ 13 kDa. That line nicely superimposes with the ζ/ζ_eq_ results of PS10k, as expected, in view of the similar *M* of the two melts. The green dashed line in Figure 3a was obtained^13^ from the data of Watanabe and co-workers^3^ on a PS melt with *M* ≈ 27 kDa in steady uniaxial elongation. Agreement with the results of PS20k and PS30k in steady shear is better at higher *S* values that characterize elongational flows.

Though the reason for the lack of universality of the ζ/ζ_eq_ vs *S* law is not clear, it may partially lie in the treatment of the glassy contribution in fast flows, the importance of which increases with decreasing *M*. As mentioned above, we have here assumed, for simplicity, that the glassy times in Table 1 reduce proportionally to ζ/ζ_eq_, but obviously different assumptions may also be explored. Indeed, the parallel superposition experiments of Matsumiya et al.^25^ clearly show that flow changes the “shape” of the glassy relaxation time spectrum, rather than simply shifting it in time.

The lack of universality of ζ­(*S*) may also be due to a violation of the fluctuation–dissipation theorem in fast flows.^17,27–30^ However, recent Brownian simulations of entangled melts show that this violation appears to have a minor effect on predictions of nonlinear rheology,^31^ even though a more systematic analysis is necessary.

Figure 4 compares the available ψ_1_ data (only referring to PS20k and PS30k) with predictions obtained from our simulations by using the reduced friction coefficient ζ reported in Figure 3. The agreement between predictions and data can certainly be deemed satisfactory, though ψ_1_ values from simulations are slightly higher than those from experiments. As expected, simulations still predict *N*_2_ ≈ 0 since friction is assumed to remain isotropic, despite its reduction. Indeed, anisotropic friction models predict a nonzero *N*_2_,^32^ but we leave this aspect to future work.

**Figure 4:**
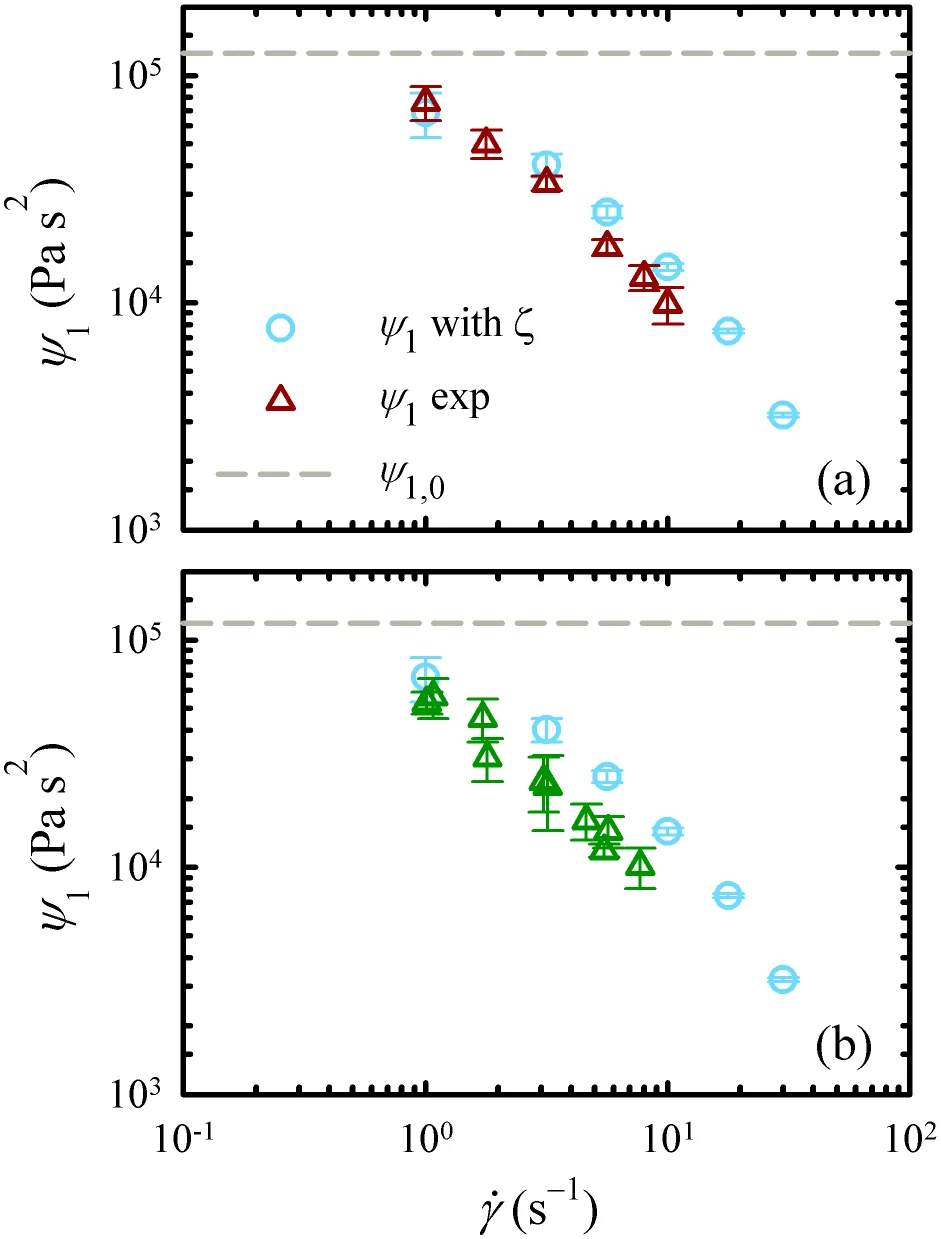
First normal stress coefficient of PS20k at 125 °C (a) and PS30k at 130 °C (b). Triangles indicate data from Dalne et al.^4^ Empty circles are predictions with the friction coefficients ζ reported in Figure 3. Dashed lines represent the zero-shear first normal stress coefficients ψ_1,0_ estimated from experimental data.

## 4 Data vs Many-Chain Simulations

The somewhat unsatisfactory comparison of single-chain simulations with data in the nonlinear range indicates that multichain effects other than friction play some role in fast flows. It appears, therefore, to be useful to perform a direct comparison with many-chain simulations.

As mentioned in the introduction, Xu et al.^21^ performed steady-shear simulations of several Kremer–Grest (KG) melts, both in the unentangled and in the entangled regimes. Their KG melts with *N* = 30 and *N* = 50 beads (N30 and N50, respectively) are characterized by a number *N_K_* of Kuhn segments and by an entanglement number *Z* comparable to those of PS20k and PS30k, respectively, as detailed below.

We determine *N_K_* for N30 and N50 by using the following definitions of the average square chain end-to-end distance $R_{ete}^{2}$ and of the chain curvilinear length *L*: $R_{ete}^{2}=N_{K}l_{K}^{2}$ and *L* = *N_K_l_K_*.^11^ The curvilinear length *L* is given by *L* = (*N* – 1)*l*, with *l* = 0.965 being the bond length of the KG chain,^33^ while $R_{ete}^{2}$ measured by Xu et al.^21^ is reported in Table 3. The resulting value of *l_K_* ≈ 1.7 agrees with literature results.^33^ The values of *N_K_*, rounded to the closest integer, are 17 and 28 for N30 and N50, respectively (Table 3). Those values are comparable to the corresponding *N_K_* values of PS20k and PS30k, equal to 21 and 32, respectively.

**Table 3:** Properties of the Two Unentangled KG Melts of Xu et al.^21^

Sample	*N*	$R_{ete}^{2}$ (LJ units)	*N_K_*
N30	30	46.3	17
N50	50	80.2	28

Concerning entanglement numbers, we know that the entanglement bead number *N_e_* of KG melts (at the standard 0.85 density) is *N_e_* ≈ 35.^33^ Thus, the number of entanglements *Z* = *N*/*N_e_* for N30 and N50 are approximately equal to 0.86 and 1.4, respectively. If the classical entanglement molar mass value *M_e_* ≈ 13kDa^11^ is used, the *Z* values for PS20k and PS30k are 1.4 and 2.2, respectively, which are slightly larger than those for N30 and N50. A better match of both *N_K_* and *Z* could be achieved by tuning KG simulation parameters, such as density and/or bending potential,^34^ but this is outside the scope of the present paper.

Figure 5 shows the steady shear viscosity of N30 and N50 (normalized with their respective zero-shear values), together with all viscosity data reported by Dalne et al.,^4^ as a function of the Rouse-time-based Weissenberg number. The Rouse times of N30 and N50 were measured by Xu et al.^21^ from the end-to-end vector autocorrelation function (see Table 1 of Xu et al.^21^). KG melts shear-thin similarly to PS, though at intermediate *Wi_R_* N30 exhibits a slightly less pronounced thinning compared to that of N50 and the PS melts. As emphasized by Xu et al., shear thinning is more “severe” in KG melts with a larger mass due to the approach to the entangled regime. No significant molar mass effects are visible in PS melts, even if PS30k is in the unentangled-to-entangled transition region. We finally note that KG simulations show a change in the slope at very high *Wi_R_* values that are not experimentally accessible.

**Figure 5:**
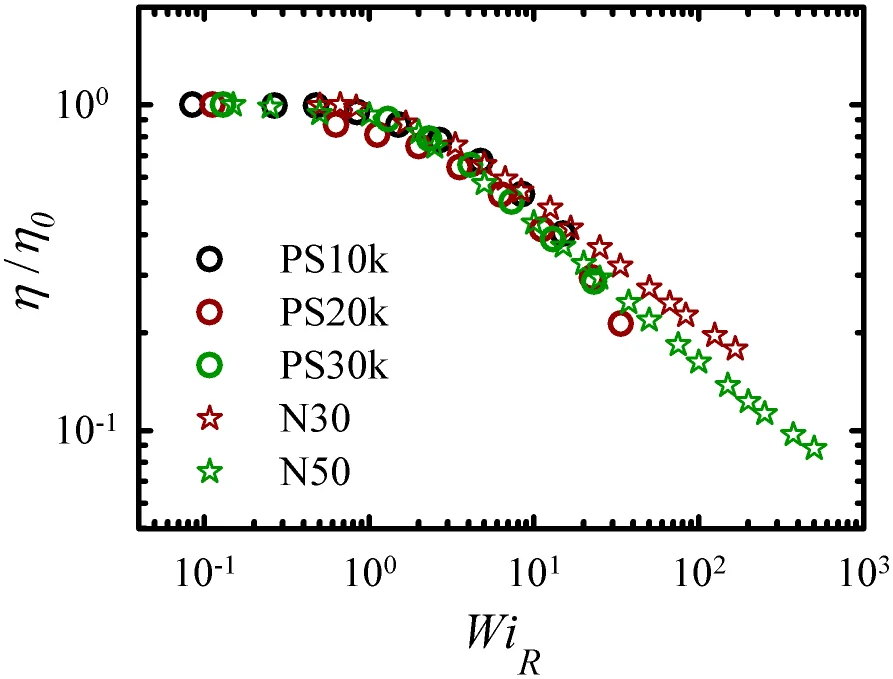
Normalized steady-shear viscosity of the PS melts from Dalne et al.^4^ (circles) and of the unentangled KG melts from Xu et al.^21^ (stars).

In Figure 6, we report the first normal stress coefficient ψ_1_, normalized with its zero-shear value ψ_1,0_. PS and KG melts show a similar thinning of the ψ_1_/ψ_1,0_ ratio at intermediate shear rates, with a slope that remains unchanged at the higher shear rates explored in the simulations. The vertical discrepancy between experiments and simulations is probably due to an overestimation of the experimental ψ_1,0_, which is not measured directly but extracted from the linear response (ψ_1,0_ = 2*G*′(ω)/ω^2^|_ω→0_).^35^ We finally note that no dependency of ψ_1_/ψ_1,0_ on molar mass is observed.

**Figure 6:**
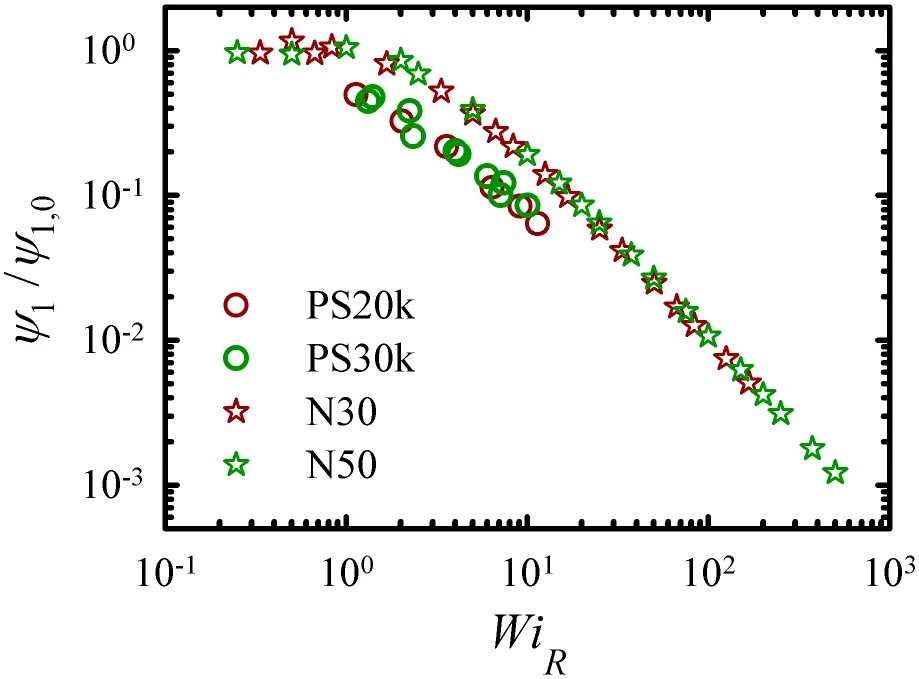
Normalized first normal stress coefficient of the PS melts from Dalne et al.^4^ (circles) and of the unentangled KG melts from Xu et al.^21^ (stars).

Figure 7 reports the ratio between the second and the first normal stress differences, −*N*_2_/*N*_1_, as a function of *Wi_R_*. Even though uncertainties at intermediate shear rates are quite significant, we can safely deduce that experiments systematically lie above simulations. In particular, PS melts exhibit a ratio −*N*_2_/*N*_1_ ≈ 0.2 ÷ 0.3, while for KG melts, it is −*N*_2_/*N*_1_ ≈ 0.1. No clear effect of both molecular weight and shear rate is evident in the experimentally explored shear rate range (*Wi_R_* ≲ 10), while in faster flows (only accessible by simulations), N30 reaches values of −*N*_2_/*N*_1_ larger than N50.

**Figure 7:**
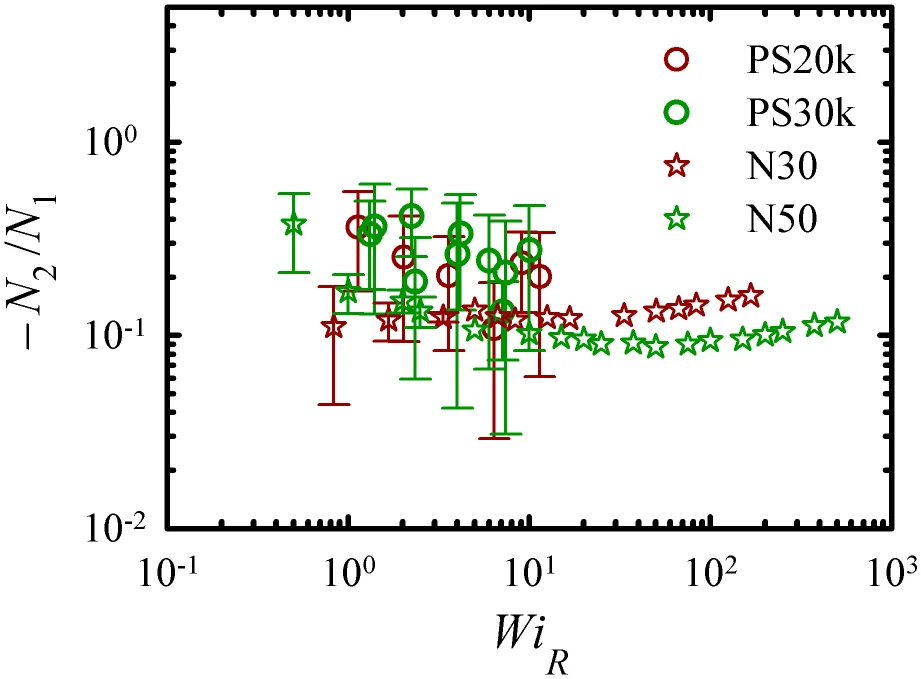
Normal stress difference ratio of the PS melts from Dalne et al.^4^ (circles) and of the unentangled KG melts from Xu et al.^21^ (stars).

The good agreement between multichain simulations of KG melts (made up by coarse-grained smooth spherical beads) and PS experiments highlights the limited effect of chemistry in shear flows. As emphasized by Watanabe and coworkers,^3,10^ the picture is significantly different in uniaxial extensional flows. Figure 8 reports extensional viscosity data^3^ for two unentangled PS and poly­(*p*-*tert*-butylstyrene) (PtBS) melts with a number of Kuhn segments and entanglements comparable to those of PS30k. Starting from *Wi_R_* ≈ 1, the two melts thicken in a similar manner because of flow-induced chain stretch. Thickening is then followed by thinning, much more pronounced in the PS melt and attributed by Watanabe and coworkers to flow-induced friction reduction effects. A similar behavior (thickening followed by thinning) was observed by Jiang^36^ in a KG melt (see Figure 8) specifically tuned to replicate Kuhn-scale features (such as *N_K_* and the Kuhn segment density υ_K_) of the PS and PtBS samples. The three different chemistries (PS, PtBS, and KG) exhibit significantly different behavior in fast extensional flows (*Wi_R_* ≳ 1), as opposed to the shear case. As also discussed by Ianniruberto et al.,^37^ this is presumably due to the different role of flow-induced friction reduction effects in the different chemistries, only emerging in the highly aligned state induced by fast extensional flows. The similarity of the nonlinear shear response (*Wi_R_* ≳ 1) of KG and PS melts is due to the rotational character of shear flows, where tumbling mitigates chain stretch (i.e., monomer coalignment) even at the highest shear rates explored by Dalne et al.^4^

**Figure 8:**
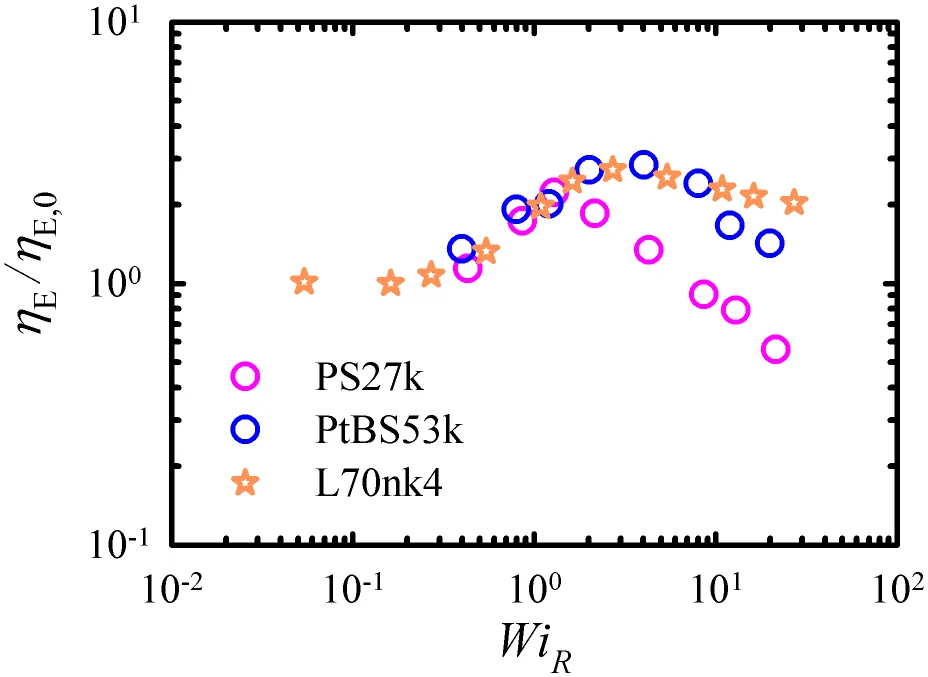
Normalized steady-state uniaxial extensional viscosity of three unentangled melts with similar *N_K_* and υ_K_: PS (pink circles), PtBS (blue circles), and KG (stars).

The difference in monomer coalignment between shear and elongational flows is clearly shown in Figure 9a. At all *Wi*_*R*_, the Kuhn segment order parameter of the sample undergoing uniaxial extension (PS27k) is significantly higher than that of the samples undergoing shear (PS20k and PS30k). Consequently, the friction coefficient in the uniaxial extensional flows of PS27k decreases more steeply with increasing *Wi*_*R*_, as confirmed by Figure 9b.

**Figure 9:**
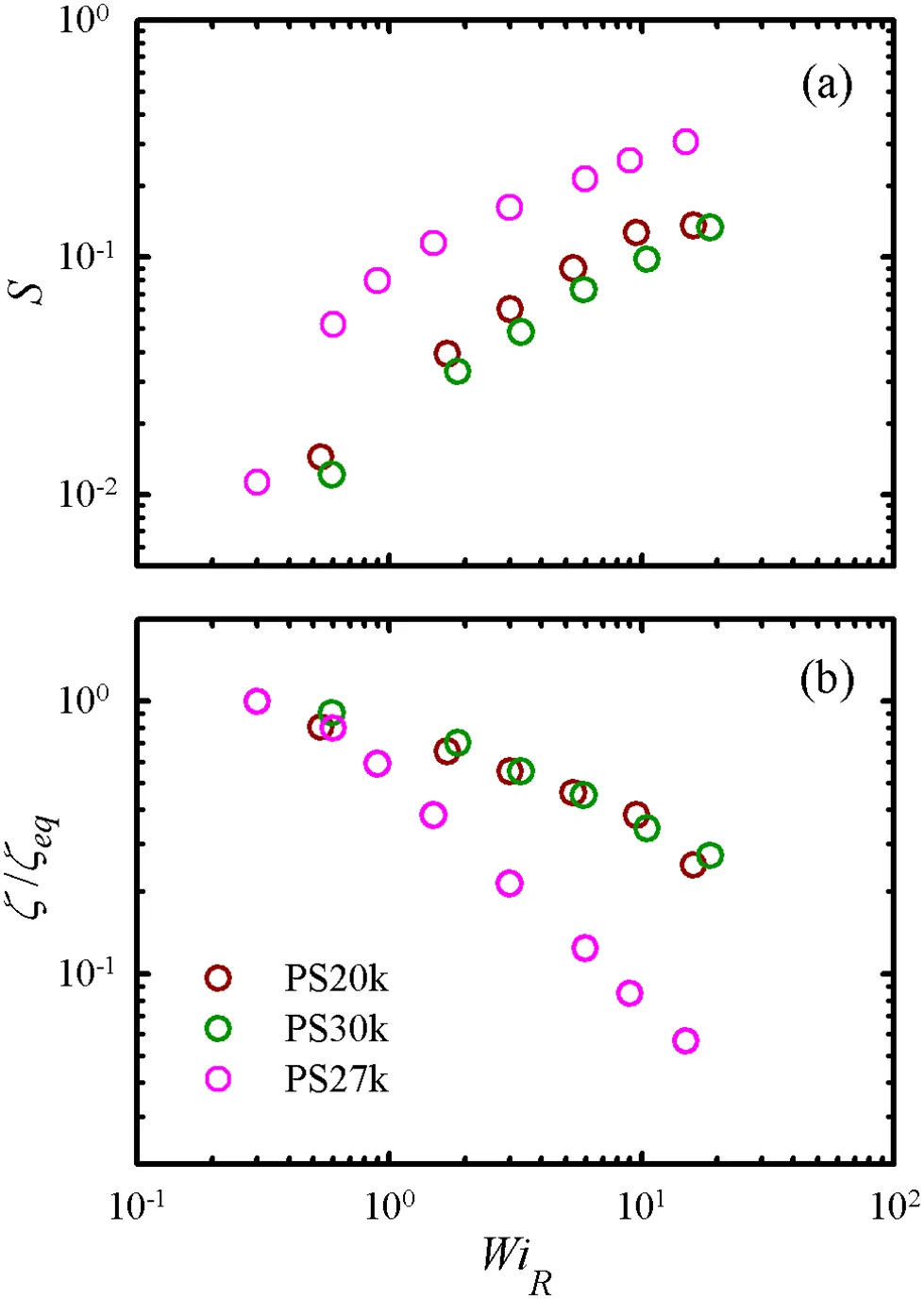
Order parameter (a) and normalized Kuhn segment friction coefficient (b) as a function of the Rouse-time-based Weissenberg number for PS melts in shear (PS20k & PS30k) and uniaxial elongational (PS27k) flows.

Finally, going back to Figure 8, we note that PS and KG results in uniaxial extension start to differ significantly at *Wi*_R_≈ 1. At this rate, it is *S*≈ 0.1 for PS27k (see Figure 9a). Such a value of *S* is barely reached by PS20k and PS30k at the highest shear rates. It seems, therefore, reasonable that we could not see any effect of chemistry in the comparison between PS and KG data in shear flows (see Figures 5–7). Differences between the two chemistries in shear flows may become apparent if PS data at higher shear rates were to become available in the future.

## 5 Concluding Remarks

We have here attempted a quantitative comparison between the very recent shear data of Dalne et al.^4^ on three unentangled PS melts and Brownian dynamics simulations of Fraenkel chains. As already shown by Ianniruberto and Marrucci for PS13k,^14^ it is here confirmed that finite extensibility is not sufficient to describe the observed shear thinning, and flow-induced friction reduction effects can be invoked to capture steady-shear viscosity data. The Kuhn segment friction coefficient comes out to be a unique function of the Kuhn segment order parameter, ζ­(*S*), for PS20k and PS30k. That function is also consistent with that applying to a PS melt with similar molar mass (PS27k) in uniaxial extensional flows.^13^ Differences in ζ­(*S*) arise for PS10k (and PS13k). This unexpected result could stem from the inaccurate treatment of the glassy response under flow and/or from a possible violation of the fluctuation–dissipation theorem in nonequilibrium conditions.

The first normal stress difference (only measured for PS20k and PS30k) was quantitatively predicted without additional adjustable parameters, while the second normal stress difference was predicted to be zero, contrary to data showing −*N*_2_/*N*_1_ ≈ 0.25, a value unexpectedly comparable to entangled melts where topological effects are important.

To better understand discrepancies between our single-chain simulations and the data of Dalne et al.,^4^ we compared those data with the only other piece of information available, i.e., the Kremer–Grest molecular dynamics simulations. The comparison was made between couples of “samples” with a similar number of Kuhn segments and entanglements, the latter being ≲ 2. PS and KG melts were shown to behave very similarly in fast shear flows, with comparable (nondimensional) values of *η*,*N*_1_,*N*_2_. We emphasize, however, that this similarity is not observed in fast uniaxial extensional flows, where chemistry-dependent flow-induced friction reduction effects are known to play a much more significant role.^3,36^

The strong proof of the existence of relatively large values of *N*_2_ in shear flows of unentangled melts indicates that single-chain simulations miss some important multichain effect(s). In particular, we plan to account for a flow/deformation-induced anisotropy of Kuhn segment mobility, as already suggested by several authors^29,30,38^ but never implemented to quantitatively describe the nonlinear rheology of unentangled melts.

Finally, future work could also aim at investigating another possible source of shear thinning in polymer melts, suggested by Jiang^39^ and based on the flow-induced truncation of the interactions between distant monomers along the same chain,^40^ which is typically ignored in polymer melts.
